# Chronic Post-Traumatic Organized Hematoma of the Brachial Artery: The Role of Multimodal Ultrasound

**DOI:** 10.3390/diagnostics16213554

**Published:** 2026-10-28

**Authors:** Zhuoyuan Li, Ziyin Liu, Tan Li

**Affiliations:** 1 Department of Cardiovascular Ultrasound, the First Hospital of China Medical University, No. 155 Nanjing Bei Street, Heping District, Shenyang 110001, China; 2 Clinical Medical Research Center of Imaging in Liaoning Province, the First Hospital of China Medical University, Shenyang 110001, China

**Keywords:** brachial artery, contrast-enhanced ultrasound, organized hematoma, pseudoaneurysm, superb microvascular imaging, traumatic vascular injury

## Abstract

Chronic post-traumatic organized hematoma of the upper extremity is uncommon and may mimic a soft-tissue mass or a pseudoaneurysm when predominantly thrombosed. We report a 60-year-old woman with progressive right upper-arm swelling and finger numbness 30 years after blunt trauma. CT angiography demonstrated a low-attenuation mass along the brachial–ulnar arterial course with tortuous vessels along its margin. Gray-scale ultrasound demonstrated a large heterogeneous, predominantly hypoechoic lesion adjacent to the brachial artery, while conventional color Doppler provided limited vascular information. Superb microvascular imaging (SMI) detected sparse intralesional and perilesional arterial flow, and contrast-enhanced ultrasound (CEUS) further demonstrated partial microbubble filling supplied by small arterial branches arising from the brachial artery. These findings initially raised the possibility of a chronic partially thrombosed pseudoaneurysm; however, no pseudoaneurysmal neck or characteristic to-and-fro flow was identified. The lesion was therefore considered most consistent with a chronic post-traumatic organized hematoma with neovascularization supplied by small brachial artery branches. Ultrasound-guided biopsy revealed organized fibrous tissue with hemorrhage and sparse inflammatory cells, supporting an organizing hematoma and excluding malignancy. This case underscores the diagnostic challenge of chronic post-traumatic organized hematoma and highlights the role of comprehensive ultrasound, particularly SMI and CEUS, in characterizing vascularity and informing clinical decisions.

**Figure 1:**
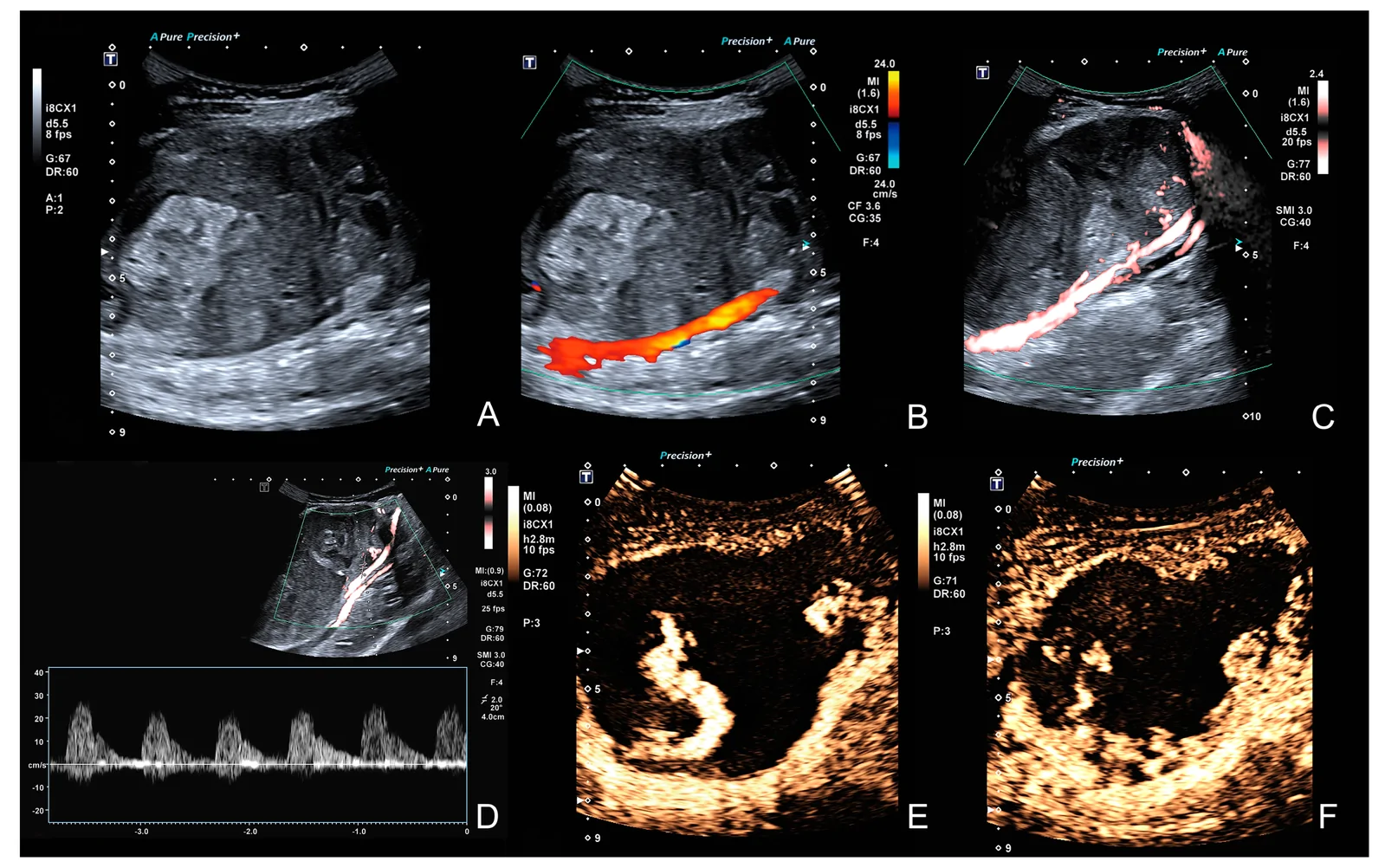
**Multimodal ultrasound findings of a chronic post-traumatic organized hematoma in the brachial artery region.** A 60-year-old woman presented with progressive right upper-arm swelling and finger numbness for 18 months. She had a remote history of blunt trauma to the right upper limb 30 years earlier, with no prior surgery, catheterization, infection, systemic disease, or connective tissue disorder. Physical examination showed marked medial upper-arm swelling, preserved distal pulses, and decreased sensorimotor function of the right fingertips. CT angiography demonstrated a low-attenuation mass along the brachial–ulnar arterial course, with tortuous vessels coursing along its margin. (**A**) Gray-scale ultrasound identified a well-defined, irregularly shaped, predominantly hypoechoic mass (15.2 × 6.6 cm) located anterior to the brachial artery, containing heterogeneous organized material. (**B**) Color Doppler imaging showed the brachial artery running adjacent to the mass with mild compression. (**C**) SMI detected sparse blood flow signals inside and around the lesion, including small arterial branches extending from the brachial system. (**D**) Spectral Doppler of these branches confirmed arterial waveforms, but no characteristic to-and-fro flow was recorded. (**E**,**F**) CEUS revealed partial microbubble filling within the hypoechoic region, originating from small arterial branches arising from the anterior wall of the brachial artery. Ultrasound examinations were performed using an Aplio i900 ultrasound system (Canon Medical Systems, Tochigi, Japan) equipped with an i8CX1 transducer. Based on these findings, the lesion is suspected to be a chronic post-traumatic organized hematoma with neovascularization supplied by small brachial arterial branches.

**Figure 2:**
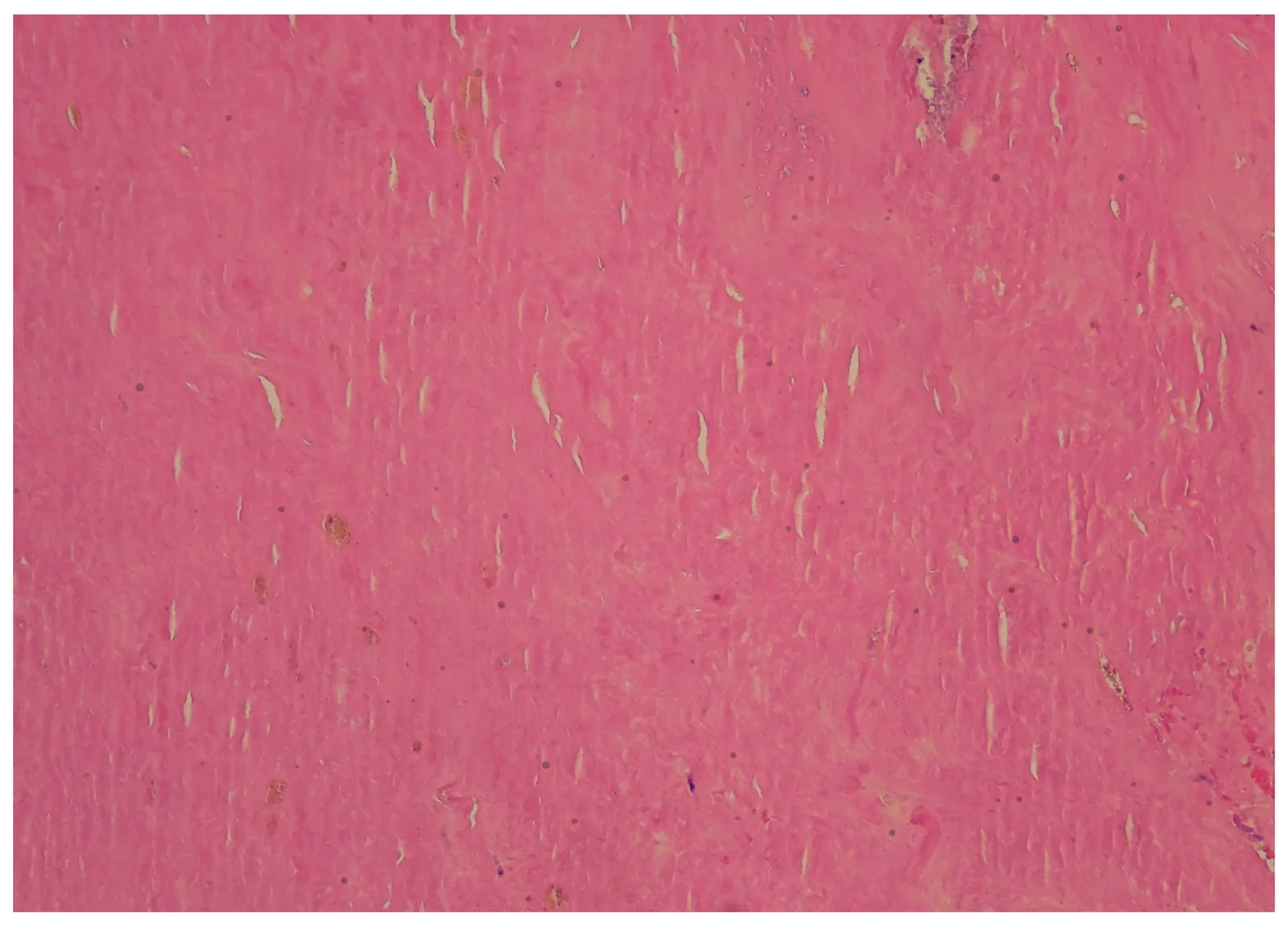
**Histopathological findings of the lesion.** With the primary aim of excluding malignancy, ultrasound-guided biopsy was performed after multidisciplinary clinical assessment, under real-time ultrasound guidance, with the needle path directed away from detectable perilesional arterial signals. Histopathological examination revealed organized fibrous tissue with hemorrhage and sparse inflammatory cells, supporting an organizing hematoma and excluding malignancy. The pathological findings, together with the long-standing history of trauma and the multimodal ultrasound evidence of residual arterial perfusion from small brachial artery branches, supported the diagnosis of a chronic post-traumatic organized hematoma. Given the potential risks of surgical intervention, the patient declined surgery and opted for observation alone. Chronic post-traumatic organized hematoma of the upper extremity is an uncommon vascular-related condition, especially when it presents decades after the initial injury and derives sparse blood supply from small arterial branches [1,2]. Multimodal ultrasound is the primary noninvasive tool for characterizing the lesion’s morphology, content, vascular origin, and anatomical relationship [3,4]. Precise identification of subtle arterial communication and intralesional flow is pivotal for differentiating this entity from a patent pseudoaneurysm and common soft-tissue mimics, thereby guiding individualized management [5]. This case highlights the critical role of comprehensive ultrasound, particularly SMI and CEUS, in achieving an accurate diagnosis and informing clinical decisions [6,7,8,9].

## Data Availability

No new data were created or analyzed in this study. The data supporting the findings of this case are not publicly available due to patient privacy and confidentiality considerations.
